# Association between prenatal provision of lipid‐based nutrient supplements and caesarean delivery: Findings from a randomised controlled trial in Malawi

**DOI:** 10.1111/mcn.13414

**Published:** 2022-07-31

**Authors:** Meeri Salenius, Juha Pyykkö, Ulla Ashorn, Kathryn G. Dewey, Austrida Gondwe, Ulla Harjunmaa, Kenneth Maleta, Minyanga Nkhoma, Stephen A. Vosti, Per Ashorn, Laura Adubra

**Affiliations:** ^1^ Center for Child, Adolescent and Maternal Health Research, Faculty of Medicine and Health Technology Tampere University Tampere Finland; ^2^ Institute for Global Nutrition and Department of Nutrition University of California Davis Davis California USA; ^3^ School of Public Health and Family Medicine, College of Medicine University of Malawi Blantyre Malawi; ^4^ Department of Paediatrics Tampere University Hospital Tampere Finland

**Keywords:** cesarian section, delivery complications, lipid‐based nutrient supplements, Malawi

## Abstract

In populations with a high prevalence of childhood and adolescent undernutrition, supplementation during pregnancy aiming at improving maternal nutritional status and preventing fetal growth restriction might theoretically lead to cephalopelvic disproportion and delivery complications. We investigated whether the prenatal provision of small‐quantity lipid‐based nutrient supplements (SQ‐LNS) was associated with an increased risk of caesarean section (CS) or other delivery complications. Pregnant Malawian women were randomised to receive daily i) iron–folic acid (IFA) capsule (control), ii) multiple micronutrient (MMN) capsule of 18 micronutrients (second control), or iii) SQ‐LNS with similar micronutrients as MMN, plus four minerals and macronutrients contributing 118 kcal. We analysed the associations of SQ‐LNS, CS, and other delivery complications using log‐binomial regressions. Among 1391 women enrolled, 1255 had delivery information available. The incidence of CS and delivery complications was 6.3% and 8.2%, respectively. The incidence of CS was 4.0%, 6.0%, and 8.9% (*p* = 0.017) in the IFA, MMN, and LNS groups, respectively. Compared to the IFA group, the relative risk (95% confidence interval) of CS was 2.2 (1.3–3.8) (*p* = 0.006) in the LNS group and 1.5 (0.8–2.7) (*p* = 0.200) in the MMN group. We found no significant differences for other delivery complications. Provision of SQ‐LNS to pregnant women may have increased the incidence of CS. The baseline rate was, however, lower than recommended. It is unclear if the higher CS incidence in the SQ‐LNS group resulted from increased obstetric needs or more active health seeking and a better supply of services. Trial registered at clinicaltrials.gov, NCT01239693.

## Key messages

- Provision of lipid‐based nutrient supplements (LNS) to pregnant Malawian women was associated with a higher incidence of caesarean delivery compared with iron–folic acid, which is the standard of care.
- The overall rate of caesarean section in this study was lower than recommended, and it remains unclear if the increased incidence in the LNS group resulted from increased obstetric needs or more active health seeking and a better supply of services.
- The association of LNS with higher caesarean delivery risk is a possible phenomenon in some settings and calls for monitoring obstetric complications in future prenatal nutritional supplementation studies and programs.

## Graphical abstract

Provision of lipid‐based nutrient supplements (LNS) to pregnant Malawian women was associated with a higher incidence of caesarean delivery compared with iron–folic acid (IFA), which is the standard of care. The overall rate of caesarean section in this study was lower than recommended, and it remains unclear if the increased incidence in the LNS group resulted from increased obstetric needs or more active health seeking and a better supply of services. The association of LNSs with higher caesarean delivery risk is a possible phenomenon in some settings and calls for monitoring obstetric complications in future prenatal nutritional supplementation studies and programs.

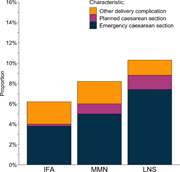

## 1 INTRODUCTION

The nutritional status of women during pregnancy plays an important role in the healthy growth and development of the fetus. Adverse conditions such as nutrient deficiencies during this period can lead to deleterious outcomes including fetal growth restriction (FGR) which can result in infants being born small for gestational age (SGA) and with low birth weight (LBW) (UNICEF, 2019). Estimated to affect around 10% of pregnancies globally (Gordijn et al., 2018), FGR is common in sub‐Saharan Africa and Southern Asia (Black et al., 2013; Dewey & Begum, 2011). Infants with FGR have increased morbidity and mortality (Colella et al., 2018; de Onis & Branca, 2016), and SGA and LBW are the leading risks for stunted growth (Danaei et al., 2016).

The first 1000 days of life, a critical time frame for human growth and development (de Onis & Branca, 2016; Martorell & Zongrone, 2012), offers a unique window of opportunity where mothers and young children have the highest potential to benefit from interventions promoting healthy growth (Dewey & Begum, 2011). Supplementing undernourished and stunted pregnant women with multiple micronutrients (MMNs) or energy has been shown to reduce the incidence of FGR (Black et al., 2013). By providing a range of key micronutrients, energy, protein, and essential fatty acids, prenatal supplementation with lipid‐based nutrient supplements (LNSs) can play an important role in improving infant and child outcomes through the prevention of FGR (Adu‐Afarwuah et al., 2007; Mangani et al., 2015). The past decade has seen a rise in research evaluating the efficacy and effectiveness of these supplements in resource‐constrained settings. The findings from several reviews suggested that LNS within the context of complementary feeding compared to no intervention is effective at improving growth outcomes (Das et al., 2019; Dewey et al., 2021), and that compared to iron–folic acid (IFA), LNS supplementation during pregnancy improves birth weight and birth length and reduces SGA and newborn stunting (Das et al., 2018). However, there is also a possible concern that improved fetal growth could cause a disproportion between the size of the fetal head and the birth canal (cephalopelvic disproportion, CPD) (Dewey & Begum, 2011). Such a scenario could lead to labour complications and the need for surgical delivery interventions (Martorell & Zongrone, 2012; Merchant et al., 2001), especially among short mothers who already have an increased risk of delivery by caesarean section (CS) (Dujardin et al., 1996; Toh‐Adam et al., 2012).

In the current study, using data from the International Lipid‐Based Nutrient Supplements (iLiNS)‐DYAD‐M trial in Malawi, we compared the incidence of CS and other delivery complications in pregnant women who received small‐quantity (SQ)‐LNSs and two control groups in which pregnant women received capsules containing either IFA or MMN. Our main hypothesis was that women receiving SQ‐LNS would have a higher incidence of CS (planned or emergency) or other delivery complications.

## 2 SUBJECTS AND METHODS

### 2.1 Study design

This was a secondary analysis of data prospectively collected as part of a dietary intervention trial, iLiNS‐DYAD‐M, in Malawi (ClinicalTrials.gov, Identifier NCT01239693), in which mother–child pairs in the intervention group received SQ‐LNS whereas mothers in the control groups received either IFA or MMN. The main outcomes of the study included birth weight, newborn length, and length for age *Z*‐score (LAZ). In the current study, we analysed the association between maternal SQ‐LNS supplementation, compared to IFA and MMN, and the incidence of CS and other delivery complications.

### 2.2 Study site and participants

The enrolment in the study took place in one public district hospital (Mangochi), one semiprivate hospital (Malindi), and two public health centres (Lungwena and Namwera) in Mangochi district, Southern Malawi. In total, the clinics provided health care to approximately 190,000 people. Recruitment for the trial was open to pregnant women who came for antenatal care at any of the clinics and met the following criteria: ultrasound‐confirmed pregnancy of under 20 completed gestation weeks, at least 15 years of age, and without any chronic health conditions.

### 2.3 Interventions

Enrolled participants were randomly assigned into three groups that were provided with daily nutrient supplements. Women in the first control group, the IFA group, received standard Malawian antenatal care, including supplementation with micronutrient capsules containing 60 mg iron and 400 μg folic acid. Women in the second control, the MMN group, received capsules that contained IFA and 16 additional micronutrients. MMN was chosen as the second control because of the benefits it might have over IFA (Smith et al., 2017). Participants in the intervention group, the LNS group, received 20 g SQ‐LNS sachets containing 118 kcal, protein, carbohydrates, essential fatty acids, sucrose, and 22 micronutrients. IFA and MMN looked and tasted identical, but the SQ‐LNS sachets looked different from the control supplements. Data collectors delivered 15 supplement doses (IFA or MMN capsules or LNS sachets) fortnightly to each participant, at their home, until delivery. There was no direct observation of the consumption of the supplements. As a measure of compliance to the interventions, at each visit, the data collectors collected any leftover supplements or empty packaging from the participants. The mean adherence to the intervention (proportion of days when the supplements were consumed) was comparable and higher than 80% in all three groups (Ashorn et al., 2015). All three groups also received intermittent preventive malaria treatment. Details of the interventions can be found elsewhere (Ashorn et al., 2015).

### 2.4 Data collection

Participants were enroled between 14 and 20 gestation weeks. At the enrolment visit, trained anthropometrists measured the participating women's weight, height, and mid‐upper arm circumference. Research nurses assessed the duration of pregnancy by measuring fetal biparietal diameter, femur length, and abdominal circumference with ultrasound imagers that used inbuilt Hadlock tables to estimate the duration of gestation. The same nurses measured the women's peripheral blood malaria parasitemia with rapid tests (Clearview Malaria Combo; British Biocell International Ltd.) and haemoglobin concentration with a finger prick. Health facility nurses tested for HIV infection in all participants, except for those who opted out or were already known to be HIV infected, by using a whole‐blood antibody rapid test (Alere Determine HIV‐1/2; Alere Medical Co., Ltd.). All participants were invited for follow‐up visits at the study clinic at 32 and 36 gestational weeks. During these visits, standardised obstetric examinations were conducted and anthropometric measurements were taken again to examine maternal weight gain during pregnancy. The mean maternal weight gain during the second and third trimesters of pregnancy was comparable between all three groups (Ashorn et al., 2017). The delivery information was collected by a clinic data collector (trained study nurses, laboratory technicians, study monitor, or study coordinator) either at the clinic or at home within 48 h after delivery (the newborn visit). A clinic data collector filled the delivery information form based on the health passport and delivery charts. We defined CS either as a planned CS or an emergency CS. In a planned CS, the woman was informed during the antenatal period that she would have to deliver by CS due to identified complications that would make vaginal delivery unsafe. In an emergency CS, the decision of the procedure was made immediately before or during labour because of a life‐threatening situation either to the mother or the child. Any delivery complication was defined as a condition of a planned CS, emergency CS, vacuum extraction, prolonged labour, large perineal tear, or symphysiotomy.

The child's length, weight, and head circumference were measured at the first clinic visit after the birth (the postnatal visit) by trained anthropometrists. We considered newborn anthropometric measurements missing if they were collected more than 6 weeks after delivery. We calculated age‐ and sex‐standardised anthropometric indices (*Z*‐scores) by using the World Health Organisation (WHO) Child Growth Standards (WHO Multicentre Growth Reference Study Group 2006). We calculated the duration of pregnancy by adding the time interval between enrolment and delivery determined by ultrasound gestational age at enrolment.

### 2.5 Sample size and statistical analysis

The sample size was originally calculated in accordance with the main objective of the iLiNS‐ DYAD‐M trial (Ashorn et al., 2015) and was based on an assumption of an effect size of at least 0.3 (difference between groups, divided by the pooled SD) for each continuous outcome, a power of 80%, and a two‐sided type I error rate of 5%. We carried out the statistical analyses with Stata 15.1 (StataCorp) based on the analysis plan written and published at ilins.org. We based the analysis on the principle of intention to treat. We excluded twin pregnancies and abortions from the analyses.

We estimated the incidence of delivery complications in the three groups and calculated relative risks (RR) for the comparison of binary endpoints. To prevent inflated type I errors caused by multiple comparisons, we used a closed testing procedure. Null hypotheses for pairwise comparisons could only be rejected if the global null hypotheses of all three groups being identical had also been rejected (Cheung, 2013).

We tested the global null hypotheses for binary endpoints either with Fisher's exact test or the log‐binomial regression model. We tested quantitative endpoints with an analysis of variance.

With the log‐binomial regression models for the binary endpoints, we used a set of Newton–Raphson maximisation of the log‐likelihood. If the algorithm failed to converge in the estimation, we used an alternative estimation algorithm with iterated reweighted least squares (Zou, 2004). With the same setting, we calculated RRs from bivariate analysis for single variables and we created cumulative stepwise multivariate log‐binomial regression models for an association attenuation analysis. For the first multivariable model, we included the child's sex, gestational age, and variables with *p* < 0.05 from bivariate analysis, excluding the child's anthropometric measurements. For the second multivariable model, we included variables that were considered intermediate outcomes (i.e., maternal weekly gestational weight gain, LAZ, weight‐for‐age *Z*‐score [WLZ], and head circumference *Z*‐score [HCZ]) with variables from the first multivariable model.

We performed likelihood ratio tests for the interaction between intervention and maternal characteristics. Maternal variables were specified in the statistical analysis plan before data analysis. Variables that were tested for interaction and analysed as stratified included maternal age, education, number of previous pregnancies, height, body mass index (BMI), alpha‐1‐acid glycoprotein, C‐reactive protein (CRP), HIV, peripheral blood malaria parasitemia, and anaemia at enrolment as well as the season of enrolment, gestational age at enrolment, food insecurity status, and child's sex. We provided stratified analyses in case of positive interaction (*p* < 0.10) or if either of the stratified comparisons of binary endpoints resulted in *p* < 0.05 and thus suggested a difference between intervention groups within a stratified subgroup. For the final analyses, each analysis was adjusted to the site of enrolment (i.e., hospitals and health clinics, to control for access to health services) and to other variables that were included in the provided stratified analyses (to control for participants with multiple classifications on the selected variables).

## 3 RESULTS

**Figure 1:**
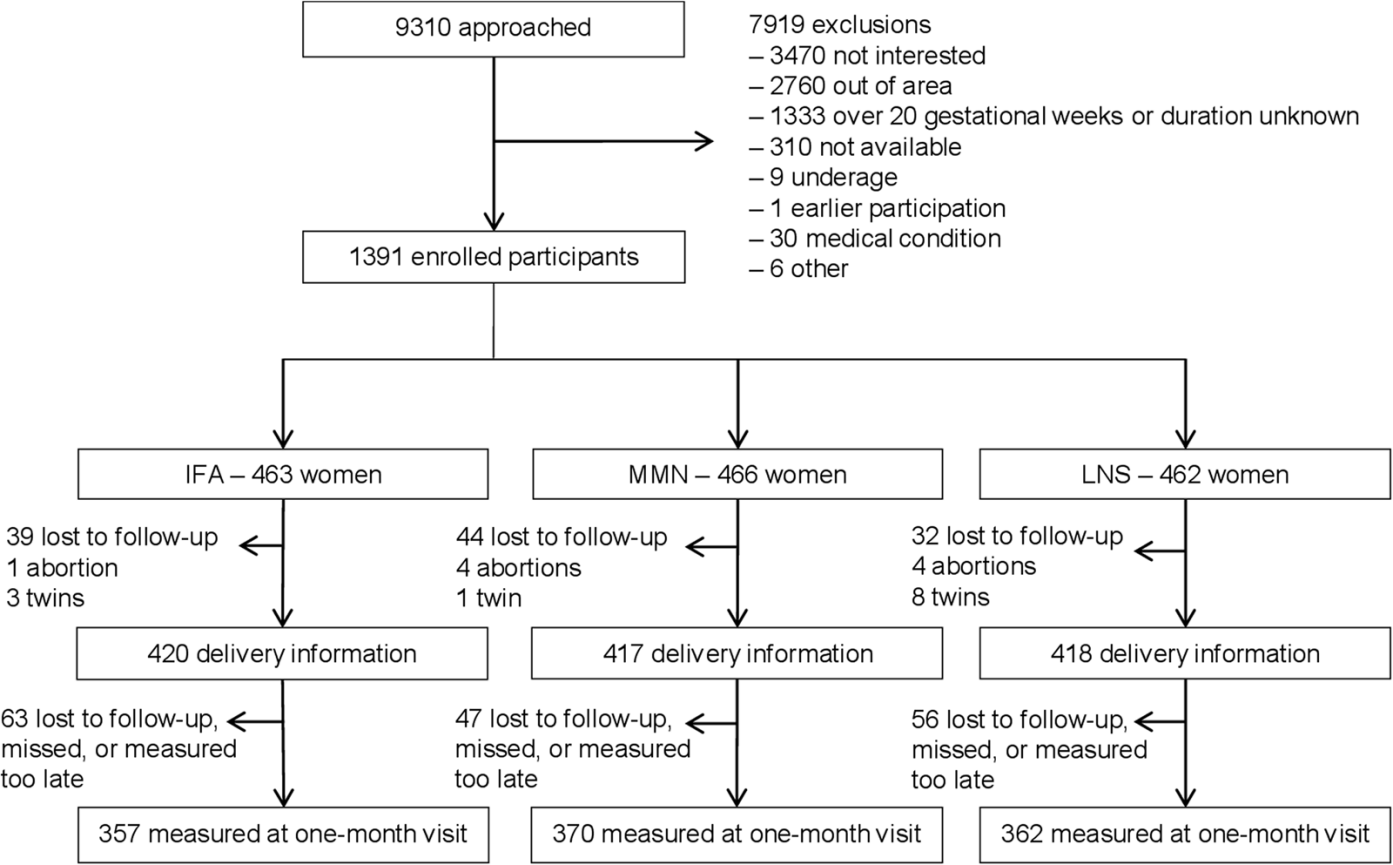
Participant flow in CONSORT recommended format. CONSORT, consolidated standards of reporting trials; IFA, iron–folic acid; LNS, lipid‐based nutrient supplement; MMN, multiple micronutrients.

Between February 2011 and August 2012, the iLiNS team enroled 1391 participants at the four study sites in southern Malawi. At enrolment, the three study groups were similar in terms of age, parity, BMI, height, and several other variables (Table 1). The delivery information was available for 1255 (90.2%) participants and anthropometric information at the post‐natal visit for 1088 infants (78.3%) (Figure 1). There was no difference in the loss to follow‐up from the enrolment to the delivery information collection between the groups (21.2%, 19.5%, and 19.9% for IFA, MMN, and LNS, respectively, *p* = 0.81). The included and excluded participants had otherwise similar characteristics except the excluded were on average younger (23 vs. 25 years, *p* < 0.001) and more likely to be primiparous (34.1% vs. 20.5%, *p* = 0.001) (Supporting Information: Table 1). Among both the included and the excluded, women in the three intervention groups had similar characteristics at baseline.

**Table 1:** Baseline characteristics of the participating women at enrolment, by study group

Characteristic	IFA	MMN	LNS
No. of participants	463	466	462
Maternal age, y	25 ± 6^a^	25 ± 6	25 ± 6
Maternal weight, kg	53.9 ± 7.4	54.0 ± 8.1	54.3 ± 8.4
Maternal height, cm	156.1 ± 5.7	156.0 ± 5.6	156.2 ± 5.7
Maternal BMI, kg/m^2^	22.1 ± 2.6	22.2 ± 2.9	22.2 ± 3.0
Gestational age at enrolment, wk	16.8 ± 2.1	16.8 ± 2.1	16.9 ± 2.2
Maternal education, completed years	3.9 ± 3.4	4.1 ± 3.4	4.1 ± 3.6
The proportion of nulliparous women, %	20.4	23.0	22.1
Proportion of anaemic women (Hb < 100 g/L), %	21.0	19.8	21.2
The proportion of women with a positive HIV test, %	15.6	11.1	14.4
The proportion of women with a positive malaria test (RDT), %	22.7	24.1	22.8

Abbreviations: BMI, body mass index; IFA, iron–folic acid; LNS, lipid‐based nutrient supplement; MMN, multiple micronutrients; RDT, rapid diagnostic test.

^a^ Mean ± SD (all such values).

The proportion (*n*) of women who reported a nonscheduled visit to a health facility during their antenatal care due to an illness, was 53.1% (223) in the IFA group, 52.5% (219) in the MMN group, and 59.6% (249) in the LNS group (*p* = 0.075). Of the 1234 women with a known site of delivery, 663 (54%) delivered in a hospital, 379 (31%) in a health centre, and 192 (16%) at home, at a traditional birth attendants facility, or elsewhere. The proportion (*n*) of hospital deliveries was 50.1% (206) in the IFA group, 53.3% (218) in the MMN group, and 57.7% (239) in the LNS group (*p* = 0.089). The proportion (*n*) of delivery at a health centre was 30.9% (127), 30.1% (123), and 31.2% (129), in the IFA, MMN, and LNS groups, respectively (*p* = 0.937).

A total of 79 (6.3%) women delivered by CS, of which 11 were planned and 68 were carried out due to an obstetric emergency. The number of delivery complications was 103 (8.2%) (Table 2). None of the mothers died during CS. Two mothers who delivered by CS and a total of eight mothers died within 6 weeks after giving birth. By study group, the incidence of CS was 4.0% in the IFA, 6.0% in the MMN, and 8.9% in the LNS group (*p* = 0.017) (Supporting Information: Figure 1). Compared to the IFA group, the RR (95% confidence interval [CI]) of CS was 2.2 (1.2, 3.8; *p* = 0.006) in the LNS group and 1.5 (0.8, 2.7; *p* = 0.20) in the MMN group (Table 2). Both emergency and planned CS were most common in the LNS group and least common in the control groups, but the differences were not statistically significant. There was a similar pattern for any delivery complication, but the RRs were not statistically significant.

**Table 2:** Comparison of delivery complications by study group

	Result by study group				Comparison between LNS and IFA group		Comparison between MMN and IFA group		Comparison between LNS and MMN group	
Characteristic	IFA (*N* = 420)	MMN (*N* = 417)	LNS (*N* = 418)	*p‐*Value^a^	Risk ratio (95% CI)	*p*‐Value^b^	Risk ratio (95% CI)	*p*‐Value^b^	Risk ratio (95% CI)	*p*‐Value^b^
Caesarean section (planned or emergency)	4.0% (17)	6.0% (25)	8.9% (37)	0.017	2.2 (1.3, 3.8)	0.006	1.5 (0.8, 2.7)	0.200	1.5 (0.9, 2.4)	0.118
Emergency caesarean section	3.8% (16)	5.0% (21)	7.4% (31)	0.071	1.9 (1.1, 3.5)	0.026	1.3 (0.7, 2.5)	0.390	1.5 (0.9, 2.5)	0.158
Planned caesarean section	0.2% (1)	1.0% (4)	1.4% (6)	0.155	6.0 (0.7, 49.9)	0.096	4.0 (0.5, 35.9)	0.212	1.5 (0.4, 5.3)	0.530
Any delivery complication^c^	6.2% (26)	8.2% (34)	10.3% (43)	0.095	1.7 (1.0, 2.7)	0.033	1.3 (0.8, 2.2)	0.273	1.3 (0.8, 1.9)	0.288

Abbreviations: CI, confidence interval; IFA, iron–folic acid; LNS, lipid‐based nutrient supplement; MMN, multiple micronutrients.

^a^ *p*‐value obtained from Fisher's exact test.

^b^ *p*‐value obtained from log‐binomial regression.

^c^ Planned caesarean section, emergency caesarean section, vacuum extraction, prolonged labour, large perineal tear, or symphysiotomy.

Table 3 shows bivariate and adjusted models for RR to CS. Besides the study intervention, CS was associated with maternal primiparity, height, education, CRP at enrolment, and with infant LAZ and WLZ at the post‐natal visit. A multivariable model adjusted for the child's sex and duration of pregnancy in addition to previously mentioned maternal variables did not change the association between the intervention and CS. Adjusting the model further with maternal weekly gestational weight gain, and infant LAZ, WLZ, and HCZ did not attenuate the association. Results were similar for emergency CS (Supporting Information: Table 2).

**Table 3:** Factors associated with caesarean section

	Bivariate analysis^a^		Multivariable model I^b^		Multivariable model II^c^	
Exposure variables	RR (95% CI)	*p*‐Value	RR (95% CI)	*p*‐Value	RR (95% CI)	*p*‐Value
Group (LNS to IFA)^d^	2.2 (1.3, 3.8)	**0.006**	2.1 (1.2, 3.8)	**0.009**	2.3 (1.2, 4.7)	**0.019**
Sex (male)	1.2 (0.8, 1.9)	0.338	1.1 (0.7, 1.8)	0.542	1.1 (0.7, 1.9)	0.674
Parity (primiparous)	2.0 (1.3, 3.1)	**0.002**	1.8 (1.1, 2.9)	**0.011**	1.9 (1.1, 3.4)	**0.018**
Gestational age (GA) at birth, wk	1.1 (1.0, 1.2)	0.236	1.1 (1.0, 1.2)	0.249	1.1 (0.9, 1.3)	0.269
Child length‐for‐age *Z*‐score (LAZ)	0.8 (0.6, 1.0)	**0.019**			0.8 (0.5, 1.1)	0.180
Child weight‐for‐age *Z*‐score	0.9 (0.7, 1.2)	0.529				
Child weight‐for‐length *Z*‐score (WLZ)	1.3 (1.0, 1.6)	**0.035**			1.1 (0.8, 1.5)	0.494
Child head circumference *Z*‐score (HCZ)	1.2 (0.9, 1.5)	0.225			1.4 (1.0, 2.0)	0.051
Maternal height, cm	0.9 (0.9, 1.0)	**<0.001**	0.9 (0.9, 1.0)	**<0.001**	0.9 (0.9, 1.0)	**<0.001**
Maternal BMI, kg/m^2^	1.1 (1.0, 1.1)	0.145				
Maternal age, y	1.0 (1.0, 1.0)	0.967				
Maternal weekly gestational weight gain, kg	0.7 (0.1, 5.4)	0.708			0.4 (0.0, 5.0)	0.489
HIV+ at enrolment	0.8 (0.4, 1.6)	0.579				
Positive malaria test (RDT) at enrolment	1.1 (0.7, 1.8)	0.639				
High AGP (>1) at enrolment	0.8 (0.4, 1.5)	0.450				
High CRP (>5) at enrolment	0.6 (0.4, 1.0)	**0.038**	0.7 (0.4, 1.0)	0.071	0.5 (0.3, 1.0)	**0.043**
Maternal education, completed years at school	1.1 (1.0, 1.1)	**0.014**	1.1 (1.0, 1.1)	**0.012**	1.1 (1.0, 1.2)	0.054
Household assets, below the median	0.7 (0.5, 1.1)	0.105				

Abbreviations: AGP, alpha‐1‐acid glycoprotein; BMI, body mass index; CI, confidence interval; CRP, C‐reactive protein; IFA, iron–folic acid; LNS, lipid‐based nutrient supplement; RDT, rapid diagnostic test; RR, relative risk.

^a^ Log‐binomial regression, nonadjusted models. Variables individually compared to caesarean section. *N* for individual variables ranged from 1049 to 1255.

^b^ Log‐binomial regression, adjusted for group, sex, primiparity, GA, maternal height, CRP, and maternal education. *N* = 1221.

^c^ Log‐binomial regression, adjusted for group, sex, primiparity, GA, maternal height, maternal weekly gestational weight gain, CRP, maternal education, LAZ, WLZ, and HCZ. *N* = 1030.

^d^ Change of one unit for continuous variables and comparison to an opposite value for binary variables.

The association between intervention and the incidence of CS was modified by maternal parity (*P‐interaction* = 0.041) and malaria parasitemia at enrolment (*P‐interaction* = 0.053) (Table 4). The RR of CS was increased in the LNS group (compared to the IFA group) among multiparous women (RR = 4.0, 95% CI: 1.8, 9.0), but there was no increased risk observed among primiparous women. Similarly, the LNS group (compared to the IFA group) had an increased risk of CS among women who were malaria negative at enrolment (RR = 3.1, 95% CI: 1.5, 6.1). There was an increased RR for CS among women with more than median education or lower than median BMI in the LNS group compared to the IFA group, but for these analyses, the formal interaction test did not yield statistically significant findings. Results were similar for emergency CS (Supporting Information: Table 3).

**Table 4:** Stratified analysis of caesarean section between groups

			Caesarean section by study group				Comparison between LNS and IFA group^a^ ^,^ ^b^		Comparison between MMN and IFA group^a^ ^,^ ^b^	
Mother's characteristic	Interaction test *p‐*value^b^	Split	IFA	MMN	LNS	*p‐*Value^c^	RR (95% CI)	*p‐*Value	RR (95% CI)	*p‐*Value
Parity	**0.041**	Primiparous	12.7% (10/79)	9.0% (8/89)	10.1% (9/89)	0.715	0.9 (0.4, 2.1)	0.733	0.7 (0.3, 1.8)	0.490
		Multiparous	2.1% (7/340)	5.2% (17/327)	8.5% (28/329)	**0.001**	4.0 (1.8, 9.0)	**0.001**	2.3 (1.0, 5.6)	0.056
Malaria	**0.053**	Malaria–	3.0% (10/330)	5.4% (17/313)	10.0% (32/319)	**0.001**	3.1 (1.5, 6.1)	**0.002**	1.6 (0.7, 3.5)	0.235
		Malaria+	7.8% (7/90)	7.8% (8/103)	5.2% (5/97)	0.737	0.7 (0.2, 2.2)	0.542	1.0 (0.4, 2.7)	0.983
Education	0.193	Education in years below median	4.3% (9/210)	5.5% (11/200)	5.4% (11/205)	0.825	1.2 (0.5, 2.8)	0.710	1.2 (0.5, 2.8)	0.716
		Education in years above or at median	3.4% (7/205)	6.1% (13/212)	11.9% (25/210)	**0.003**	3.3 (1.5, 7.6)	**0.004**	1.8 (0.7, 4.3)	0.210
BMI	0.130	BMI below median	2.3% (5/216)	6.9% (15/217)	8.9% (18/203)	**0.009**	3.6 (1.3, 9.5)	**0.011**	2.8 (1.0, 7.6)	**0.041**
		BMI above or at median	5.9% (12/204)	5.0% (10/200)	8.8% (19/215)	0.253	1.5 (0.7, 3.0)	0.285	0.8 (0.3, 1.8)	0.569

Abbreviations: BMI, body mass index; CI, confidence interval; IFA, iron–folic acid; LNS, lipid‐based nutrient supplement; MMN, multiple micronutrients; RR, relative risk.

^a^ RR and *p*‐value obtained from log‐binomial regression.

^b^ All interaction test and stratified analyses were adjusted for binary variables malaria, parity, education, BMI, and four‐categorial site (hospitals and health clinics).

^c^ *p*‐value obtained from Fisher's exact test.

## 4 DISCUSSION

In the present post‐hoc analysis study, we aimed to investigate the effect of SQ‐LNS provision to pregnant women on planned or emergency CS and any delivery complications (planned or emergency CS, vacuum extraction, prolonged labour, large perineal tear, or symphysiotomy). Among 1088 mother–infant pairs in rural Malawi, provision of SQ‐LNS to pregnant women was associated with a two‐fold incidence of CS compared with the IFA control supplement. The association of SQ‐LNS intervention and CS incidence was not mediated by the newborn size, but parity and malaria at enrolment modified it; provision of SQ‐LNS was associated with an increased proportion of CS among multiparous and malaria negative women.

Although the following challenges are often faced in trials conducted in the field context and in similar settings, internal validity in our study could have been compromised by a relatively large number of missing data, delay in anthropometric measurements of some participants, our inability to exactly observe the consumption of the supplement, the lack of information on reasons for CS, and the facts that women receiving SQ‐LNS were not blinded to their treatment and CS was not a prespecified outcome of the trial. Hence, it is possible that the association between SQ‐LNS supplementation and higher CS rate was spurious. However, most of the caesarean deliveries in this study were unplanned and the incidence of CS was nonsignificantly higher also in the properly masked MMN group. The small *p*‐value suggests that the likelihood of no difference in CS between SQ‐LNS versus the IFA control is small. Furthermore, it is not probable that loss to follow‐up would have modified the effect because even though the excluded participants were overall younger and more likely to be primiparous, these potentially confounding variables were balanced between groups. The data are, therefore, consistent with the possibility that SQ‐LNS provision increased the incidence of CS in this population.

Findings for childbirth complication outcomes in the context of supplementation programs are scarce. A recent review that explored the effects of LNS on maternal, and birth outcomes in pregnant women in low‐ and middle‐income countries found no difference between IFA versus LNS and MMN versus LNS with regard to adverse effects including hospitalisation, miscarriage, preterm delivery, and maternal deaths (Das et al., 2018). To our knowledge, only one study conducted in rural Bangladesh reported on the possible association between LNS and delivery complications (Mridha et al., 2017). The study was a secondary analysis of a trial (Mridha et al., 2016) that reported increased birth size and head circumference after LNS supplementation. Despite this positive impact, no significant effect of LNS on CS or delivery complications was found (Mridha et al., 2017); the prevalence of CS was 15.6% in the LNS group and 14.2% in the control group that received IFA. The CS rates in Bangladesh were in the WHO recommended range for CS set at 10%–15% of all births (Betran et al., 2016), whereas the overall rate of CS in our study was below it, at only 6.3%. These findings suggest that the contribution of LNS to increased rates of CS is not universal but rather a possible phenomenon, that may occur in some settings. The trend is that CS rates are increasing globally, although at a slower pace in sub‐Saharan Africa (Betrán et al., 2016). In 2020, the rate of delivery by CS in Malawi was 7.7%, whereas, in 2010, the rate was 4.6% (UNICEF Data Warehouse, 2021). If the trend continues, Sub‐Saharan Africa may face an increase in maternal morbidity and mortality associated with unmet needs and unsafe provision of CS, and concomitant overuse of the procedure (Betrán et al., 2021). Hence, in a context where a rise in the use of maternal nutrient supplementation in low‐ and middle‐income countries may be expected (Das et al., 2018), data on CS are even more essential and will play an important role in tailoring interventions and policies.

The mechanism of the possible increase in CS after maternal SQ‐LNS supplementation is unknown. An increase in fetal size could cause CPD which could lead to obstructed labour (Dewey & Begum, 2011) which we speculated to be the primary pathway of the association between LNS and CS in our study. This hypothesis was plausible given our earlier findings suggesting that LNS provision increased, though modestly and non‐significantly, mean birth weight and length (Ashorn et al., 2015). In the present study, there was, however, no reduction in the strength of association between the intervention and CS in statistical mediation analysis with newborn size. Hence, it is possible that the association between maternal SQ‐LNS provision and higher CS rate was spurious, and not causal at all. Alternatively, it could be related to more active health seeking and a better supply of obstetric services to women who were known to have received SQ‐LNS and who might thus have increased fears of childbirth complications (Clermont et al., 2018). Due to the differences in packaging, the data collectors delivering the supplements knew which mothers were receiving LNS and might have given them additional advice. The nurses who conducted the antenatal follow‐up (at 32 and 36 gestational weeks) at the study clinic were not aware of group allocation, but it is possible that the participants disclosed information on their supplements. Consequently, the nurses may have also provided additional advice to those they suspected were receiving LNS which could have led to fears about childbirth complications and to more active health seeking. The findings that a higher proportion of women in the SQ‐LNS group, than in the two control groups, delivered in a hospital and that CS prevalence was well below the WHO‐recommended level of 10%–15% even in the SQ‐LNS group, speak for increased health seeking and service provision as possible explanations. The health‐seeking possibility is further supported by the finding that women in the SQ‐LNS group made more non‐scheduled health facility visits because of an acute illness than women in the other groups.

The higher proportion of CS among multiparous but not primiparous women receiving LNS is somewhat surprising. It is noteworthy, however, that primiparous mothers had overall higher rates of CS, possibly reflecting that poor birth outcomes in first‐time pregnant mothers are not nutritionally mediated, as also speculated in Burkina Faso (Huybregts et al., 2009). Mothers infected with malaria at enrolment may also have had a reduced potential to respond to the nutritional intervention. Early malaria in pregnancy can contribute to the pathogenesis of LBW and FGR (Chua et al., 2021) through impaired placental nutrient transport due to placental insufficiency caused by parasite sequestration (Rogerson et al., 2007). In our study, infants born to malaria‐positive mothers may thus have suffered from a nutritionally worse fetal environment, resulting in a smaller birth size and therefore, a lower need for CS.

Provision of LNS to infants and young children during the complementary feeding period is an effective intervention to promote growth and development (Das et al., 2019; Dewey et al., 2021; Prado et al., 2021), improve micronutrient status (Wessells et al., 2021), and prevent malnutrition and mortality (Dewey et al., 2021; Stewart et al., 2020) in over 6‐month‐old children. This positive experience, the ease of use, and preliminary evidence of benefits from antenatal administration (Das et al., 2018) are likely to lead to increased use of LNS products also for maternal supplementation during pregnancy. However, given the findings from our study pointing to a higher risk of CS for women receiving SQ‐LNS during pregnancy, we recommend monitoring obstetric complications in future prenatal nutritional supplementation studies and programs.

## ACKNOWLEDGEMENTS

This study was funded by the Finnish Funding Agency for Technology and Innovation; the Bill & Melinda Gates Foundation through grant OPP49817 to the University of California Davis, the Office of Health, Infectious Diseases, and Nutrition, Bureau for Global Health, U.S. Agency for International Development under terms of Cooperative Agreement No. AID‐OAA‐A‐12‐00005, through the Food and Nutrition Technical Assistance III Project, managed by FHI 360; the Foundation for Paediatric Research in Finland; and the Competitive State Research Financing of the Expert Responsibility area of Tampere University Hospital. The funders had no role in study design, data collection and analysis, decision to publish, or preparation of the manuscript.

## CONFLICT OF INTEREST

The authors declare no conflict of interest.

### 1 ETHICS STATEMENT

The study was performed according to Good Clinical Practice guidelines and the ethical standards of the Helsinki Declaration. The protocol was approved by the College of Medicine Research and Ethics Committee, University of Malawi, Malawi, and the Ethical Committee of Pirkanmaa Hospital District, Finland. Only participants who signed or thumb‐printed an informed consent form were enroled in the study.

## Supporting information

Supporting information.

Supporting information.

Supporting information.

Supporting information.

## Data Availability

Data described in the manuscript will be made publicly and freely available upon publication at https://doi.org/10.5281/zenodo.5779432. Code book and analytic code are available at https://github.com/pyykkojuha/scientific_publications/tree/main/code/lns_cs.

## References

[mcn13414-bib-0001] Adu‐Afarwuah, S. , Lartey, A. , Brown, K. H. , Zlotkin, S. , Briend, A. , & Dewey, K. G. (2007). Randomized comparison of 3 types of micronutrient supplements for home fortification of complementary foods in Ghana: Effects on growth and motor development. The American Journal of Clinical Nutrition, 86, 412–420. 10.1093/ajcn/86.2.412 17684213

[mcn13414-bib-0002] Ashorn, P. , Alho, L. , Ashorn, U. , Cheung, Y. B. , Dewey, K. G. , Harjunmaa, U. , Lartey, A. , Nkhoma, M. , Phiri, N. , Phuka, J. , Vosti, S. A. , Zeilani, M. , & Maleta, K. (2015). The impact of lipid‐based nutrient supplement provision to pregnant women on newborn size in rural Malawi: A randomized controlled trial. The American Journal of Clinical Nutrition, 101(2), 387–397. 10.3945/ajcn.114.088617 25646337

[mcn13414-bib-0003] Ashorn, P. , Poelman, B. , Dewey, K. G. , Maleta, K. , Klein, N. , Rogerson, S. , & Meshnick, S. R. (2017). The impact of dietary supplementation with lipid‐based nutrient supplements on maternal health and pregnancy outcomes in rural Malawi. FHI 360/Food and Nutrition Technical Assistance III Project (FANTA), p. 148. https://www.fantaproject.org/sites/default/files/resources/FANTA-LNS-RTI-Part-I-Mar2017.pdf

[mcn13414-bib-0004] Betrán, A. P. , Torloni, M. R. , Zhang, J. J. , & Gülmezoglu, A. M. , & WHO Working Group on Caesarean Section . (2016). WHO statement on caesarean section rates. BJOG: An International Journal of Obstetrics and Gynaecology, 123, 667–670. 10.1111/1471-0528.13526 26681211 PMC5034743

[mcn13414-bib-0005] Betrán, A. P. , Ye, J. , Moller, A.‐B. , Souza, J. P. , & Zhang, J. (2021). Trends and projections of caesarean section rates: Global and regional estimates. BMJ Global Health, 6, e005671. 10.1136/bmjgh-2021-005671 PMC8208001 34130991

[mcn13414-bib-0006] Black, R. E. , Victora, C. G. , Walker, S. P. , Bhutta, Z. A. , Christian, P. , & de Onis, M. , Maternal and Child Nutrition Study Group . (2013). Maternal and child undernutrition and overweight in low‐income and middle‐income countries. Lancet, 382, 427–451. 10.1016/S0140-6736(13)60937-X 23746772

[mcn13414-bib-0007] Cheung, Y. B. (2013). Statistical analysis of human growth and development. Chapman and Hall/CRC. 10.1201/b15979

[mcn13414-bib-0008] Chua, C. L. L. , Hasang, W. , Rogerson, S. J. , & Teo, A. (2021). Poor birth outcomes in malaria in pregnancy: Recent insights into mechanisms and prevention approaches. Frontiers in Immunology, 12, 621382. 10.3389/fimmu.2021.621382 33790894 PMC8005559

[mcn13414-bib-0009] Clermont, A. , Kodish, S. R. , Matar Seck, A. , Salifou, A. , Rosen, J. , Grais, R. F. , & Isanaka, S. (2018). Acceptability and utilization of three nutritional supplements during pregnancy: Findings from a longitudinal, mixed‐methods study in Niger. Nutrients, 10, 1073. 10.3390/nu10081073 PMC6115835 30103529

[mcn13414-bib-0010] Colella, M. , Frérot, A. , Novais, A. R. B. , & Baud, O. (2018). Neonatal and long‐term consequences of fetal growth restriction. Current Pediatric Reviews, 14, 212–218. 10.2174/1573396314666180712114531 29998808 PMC6416241

[mcn13414-bib-0011] Danaei, G. , Andrews, K. G. , Sudfeld, C. R. , Fink, G. , McCoy, D. C. , Peet, E. , & Fawzi, W. W. (2016). Risk factors for childhood stunting in 137 developing countries: A comparative risk assessment analysis at global, regional, and country levels. PLoS Medicine, 13, e1002164. 10.1371/journal.pmed.1002164 27802277 PMC5089547

[mcn13414-bib-0012] Das, J. K. , Hoodbhoy, Z. , Salam, R. A. , Bhutta, A. Z. , Valenzuela‐Rubio, N. G. , Weise Prinzo, Z. , & Bhutta, Z. A. (2018). Lipid‐based nutrient supplements for maternal, birth, and infant developmental outcomes. The Cochrane Database of Systematic Reviews, 8, CD012610. 10.1002/14651858.CD012610.pub2 30168868 PMC6513224

[mcn13414-bib-0013] Das, J. K. , Salam, R. A. , Hadi, Y. B. , Sadiq Sheikh, S. , Bhutta, A. Z. , Weise Prinzo, Z. , & Bhutta, Z. A. (2019). Preventive lipid‐based nutrient supplements given with complementary foods to infants and young children 6 to 23 months of age for health, nutrition, and developmental outcomes. The Cochrane Database of Systematic Reviews, 5, CD012611. 10.1002/14651858.CD012611.pub3 31046132 PMC6497129

[mcn13414-bib-0014] de Onis, M. , & Branca, F. (2016). Childhood stunting: A global perspective. Maternal & child nutrition, 12, 12–26. 10.1111/mcn.12231 27187907 PMC5084763

[mcn13414-bib-0015] Dewey, K. G. , & Begum, K. (2011). Long‐term consequences of stunting in early life. Maternal & child nutrition, 7, 5–18. 10.1111/j.1740-8709.2011.00349.x 21929633 PMC6860846

[mcn13414-bib-0016] Dewey, K. G. , Wessells, K. R. , Arnold, C. D. , Prado, E. L. , Abbeddou, S. , Adu‐Afarwuah, S. , & Stewart, C. P. (2021). Characteristics that modify the effect of small‐quantity lipid‐based nutrient supplementation on child growth: An individual participant data meta‐analysis of randomized controlled trials. The American Journal of Clinical Nutrition, 114, 15S–42S. 10.1093/ajcn/nqab278 34590672 PMC8560308

[mcn13414-bib-0017] Dujardin, B. , Van Cutsem, R. , & Lambrechts, T. (1996). The value of maternal height as a risk factor of dystocia: A meta‐analysis. Tropical Medicine & International Health, 1, 510–521. 10.1046/j.1365-3156.1996.d01-83.x 8765460

[mcn13414-bib-0018] Gordijn, S. J. , Beune, I. M. , & Ganzevoort, W. (2018). Building consensus and standards in fetal growth restriction studies. Best Practice & Research Clinical Obstetrics & Gynaecology, 49, 117–126. 10.1016/j.bpobgyn.2018.02.002 29576470

[mcn13414-bib-0019] Huybregts, L. , Roberfroid, D. , Lanou, H. , Menten, J. , Meda, N. , Van Camp, J. , & Kolsteren, P. (2009). Prenatal food supplementation fortified with multiple micronutrients increases birth length: A randomized controlled trial in rural Burkina Faso. The American Journal of Clinical Nutrition, 90, 1593–1600. 10.3945/ajcn.2009.28253 19812173

[mcn13414-bib-0020] Mangani, C. , Maleta, K. , Phuka, J. , Cheung, Y. B. , Thakwalakwa, C. , Dewey, K. , & Ashorn, P. (2015). Effect of complementary feeding with lipid‐based nutrient supplements and corn‐soy blend on the incidence of stunting and linear growth among 6‐ to 18‐month‐old infants and children in rural Malawi. Maternal & Child Nutrition, 11, 132–143. 10.1111/mcn.12068 23795976 PMC6860208

[mcn13414-bib-0021] Martorell, R. , & Zongrone, A. (2012). Intergenerational influences on child growth and undernutrition. Paediatric and Perinatal Epidemiology, 26, 302–314. 10.1111/j.1365-3016.2012.01298.x 22742617

[mcn13414-bib-0022] Merchant, K. M. , Villar, J. , & Kestler, E. (2001). Maternal height and newborn size relative to risk of intrapartum caesarean delivery and perinatal distress. BJOG: An International Journal of Obstetrics and Gynaecology, 108, 689–696. 10.1111/j.1471-0528.2001.00181.x 11467692

[mcn13414-bib-0023] Mridha, M. K. , Matias, S. L. , Chaparro, C. M. , Paul, R. R. , Hussain, S. , Vosti, S. A. , & Dewey, K. G. (2016). Lipid‐based nutrient supplements for pregnant women reduce newborn stunting in a cluster‐randomized controlled effectiveness trial in Bangladesh. The American Journal of Clinical Nutrition, 103, 236–249. 10.3945/ajcn.115.111336 26607935 PMC6443293

[mcn13414-bib-0024] Mridha, M. K. , Matias, S. L. , Paul, R. R. , Hussain, S. , Sarker, M. , Hossain, M. , & Dewey, K. G. (2017). Prenatal Lipid‐Based nutrient supplements do not affect pregnancy or childbirth complications or cesarean delivery in Bangladesh: A cluster‐randomized controlled effectiveness trial. The Journal of Nutrition, 147, 1776–1784. 10.3945/jn.117.248880 28724657

[mcn13414-bib-0025] Prado, E. L. , Arnold, C. D. , Wessells, K. R. , Stewart, C. P. , Abbeddou, S. , Adu‐Afarwuah, S. , Arnold, B. F. , Ashorn, U. , Ashorn, P. , Becquey, E. , Brown, K. H. , Chandna, J. , Christian, P. , Dentz, H. N. , Dulience, S. J. L. , Fernald, L. C. H. , Galasso, E. , Hallamaa, L. , Hess, S. Y. , … Dewey, K. G. (2021). Small‐quantity lipid‐based nutrient supplements for children age 6–24 months: A systematic review and individual participant data meta‐analysis of effects on developmental outcomes and effect modifiers. The American Journal of Clinical Nutrition, 114, 43S–67S. 10.1093/ajcn/nqab277 34590116 PMC8560311

[mcn13414-bib-0026] Rogerson, S. J. , Hviid, L. , Duffy, P. E. , Leke, R. F. G. , & Taylor, D. W. (2007). Malaria in pregnancy: Pathogenesis and immunity. The Lancet Infectious Diseases, 7, 105–117. 10.1016/S1473-3099(07)70022-1 17251081

[mcn13414-bib-0027] Smith, E. R. , Shankar, A. H. , Wu, L. S.‐F. , Aboud, S. , Adu‐Afarwuah, S. , Ali, H. , Agustina, R. , Arifeen, S. , Ashorn, P. , Bhutta, Z. A. , Christian, P. , Devakumar, D. , Dewey, K. G. , Friis, H. , Gomo, E. , Gupta, P. , Kæstel, P. , Kolsteren, P. , Lanou, H. , … Sudfeld, C. R. (2017). Modifiers of the effect of maternal multiple micronutrient supplementation on stillbirth, birth outcomes, and infant mortality: A meta‐analysis of individual patient data from 17 randomised trials in low‐income and middle‐income countries. The Lancet. Global Health, 5, e1090–e1100. 10.1016/S2214-109X(17)30371-6 29025632

[mcn13414-bib-0028] Stewart, C. P. , Wessells, K. R. , Arnold, C. D. , Huybregts, L. , Ashorn, P. , Becquey, E. , Humphrey, J. H. , & Dewey, K. G. (2020). Lipid‐based nutrient supplements and all‐cause mortality in children 6–24 months of age: A meta‐analysis of randomized controlled trials. The American Journal of Clinical Nutrition, 111, 207–218. 10.1093/ajcn/nqz262 31697329

[mcn13414-bib-0029] Toh‐Adam, R. , Srisupundit, K. , & Tongsong, T. (2012). Short stature as an independent risk factor for cephalopelvic disproportion in a country of relatively small‐sized mothers. Archives of Gynecology and Obstetrics, 285, 1513–1516. 10.1007/s00404-011-2168-3 22187064 PMC3351595

[mcn13414-bib-0030] UNICEF . (2019). The state of the world's children. Retrieved June 23, 2021, from https://www.unicef.org/reports/state-ofworlds-children-2019

[mcn13414-bib-0031] UNICEF Data Warehouse . (2021). Cross‐sector indicator: Malawi: C‐section rate – percentage of deliveries by caesarean section. Retrieved May 22, 2022, from https://data.unicef.org/resources/data_explorer/unicef_f/?ag=UNICEF%26df=GLOBAL_DATAFLOW%26ver=1.0%26dq=MWI.MNCH_CSEC.%26startPeriod=1970%26endPeriod=2022

[mcn13414-bib-0032] Wessells, K. R. , Arnold, C. D. , Stewart, C. P. , Prado, E. L. , Abbeddou, S. , Adu‐Afarwuah, S. , Ashorn, P. , Arnold, B. F. , Ashorn, U. , Becquey, E. , Brown, K. H. , Byrd, K. A. , Campbell, R. K. , Christian, P. , Fernald, L. C. H. , Fan, Y.‐M. , Galasso, E. , Hess, S. Y. , Huybregts, L. , … Dewey, K. G. (2021). Characteristics that modify the effect of small‐quantity lipid‐based nutrient supplementation on child anemia and micronutrient status: An individual participant data meta‐analysis of randomized controlled trials. The American Journal of Clinical Nutrition, 114, 68S–94S. 10.1093/ajcn/nqab276 34590114 PMC8560313

[mcn13414-bib-0033] Zou, G. (2004). A modified Poisson regression approach to prospective studies with binary data. American Journal of Epidemiology, 159, 702–706. 10.1093/aje/kwh090 15033648

