# Supplementary figures and images for: Association between prenatal provision of lipid‐based nutrient supplements and caesarean delivery: Findings from a randomised controlled trial in Malawi
Source: Matern Child Nutr. 2022 Jul 31;18(4):e13414. doi: 10.1111/mcn.13414 (PMC9480947; doi:10.1111/mcn.13414)

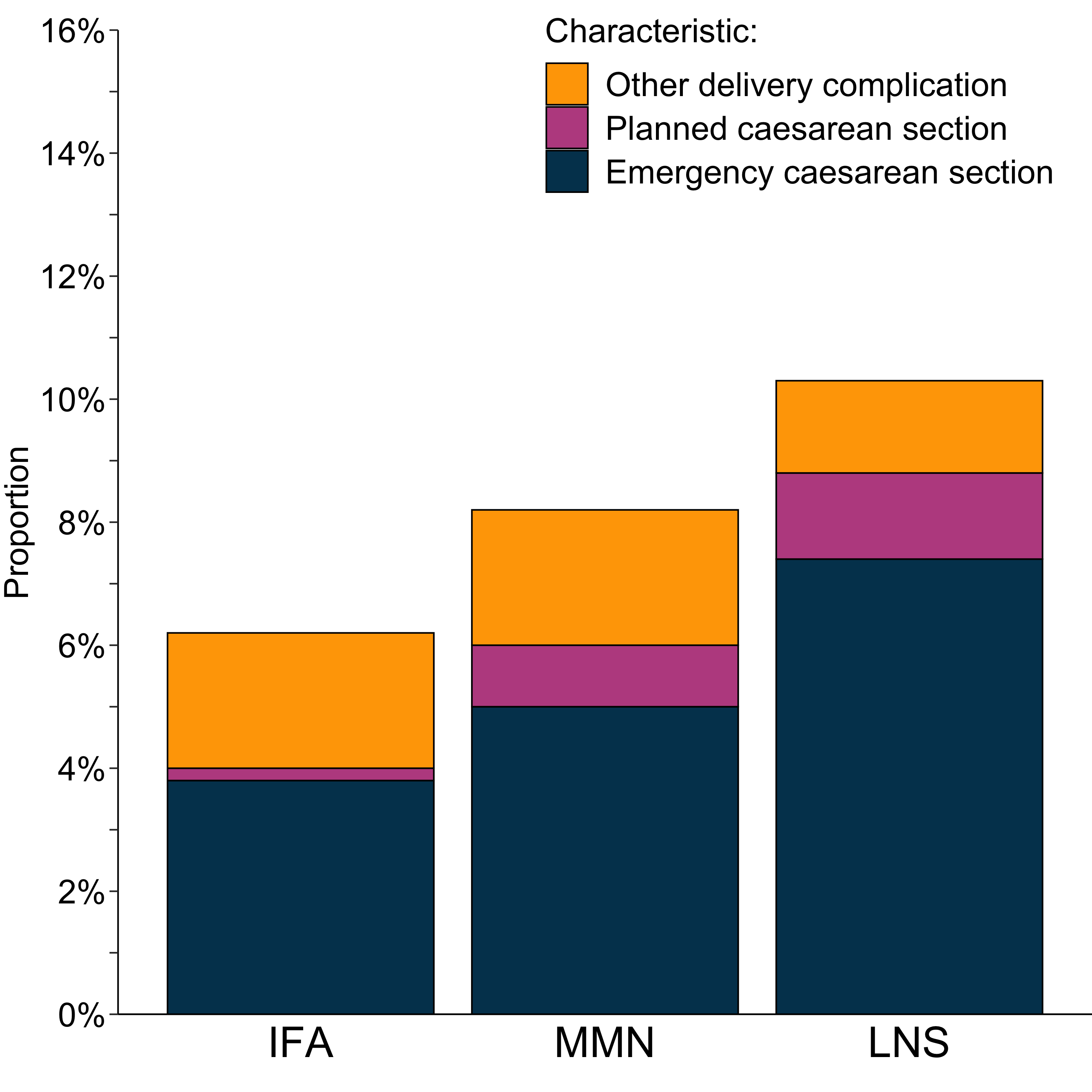

Supplement: Supplementary file 1 — Supporting information. [file MCN-18-e13414-s001.tiff]
