# Supplementary material for: Association between prenatal provision of lipid‐based nutrient supplements and caesarean delivery: Findings from a randomised controlled trial in Malawi
Source: Matern Child Nutr. 2022 Jul 31;18(4):e13414. doi: 10.1111/mcn.13414 (PMC9480947; doi:10.1111/mcn.13414)
Supplement: Supplementary file 2 — Supporting information. [file MCN-18-e13414-s004.docx]

**Supplemental Table 1.** Comparison of analyzed sample (i.e., included) to those lost to follow-up (between being enrolled and having delivery information, i.e., excluded) and baseline characteristics at enrollment of included and excluded by study groups. ^a^

|  |  |  |  | Included | | |  | Excluded | | |  |
| --- | --- | --- | --- | --- | --- | --- | --- | --- | --- | --- | --- |
| Characteristic | Included | Excluded | *P* value^b^ | IFA | MMN | LNS | *P* value^b^ | IFA | MMN | LNS | *P* value^b^ |
| No. of participants | 1255 | 136 | N/A | 420 | 417 | 418 | N/A | 43 | 49 | 44 | N/A |
| Maternal age, y | 25 ± 6^c^ | 23 ± 5 | **<0.001** | 25 ± 6 | 25 ± 6 | 25 ± 6 | 0.93 | 22 ± 5 | 23 ± 6 | 25 ± 6 | 0.07 |
| Maternal weight, kg | 53.9 ± 7.9 | 55.4 ± 8.3 | **0.035** | 53.9 ± 7.5 | 53.9 ± 8.3 | 54.0 ± 8.0 | 0.98 | 53.7 ± 6.4 | 55.0 ± 6.5 | 57.6 ± 11.0 | 0.08 |
| Maternal height, cm | 156.1 ± 5.7 | 155.7 ± 5.4 | 0.38 | 156.2 ± 5.7 | 156.0 ± 5.7 | 156.1 ± 5.7 | 0.91 | 155.2 ± 4.9 | 155.4 ± 5.4 | 156.3 ± 5.8 | 0.62 |
| Maternal BMI, kg/m^2^ | 22.1 ± 2.8 | 22.8 ± 2.9 | **0.006** | 22.1 ± 2.6 | 22.1 ± 2.9 | 22.1 ± 2.8 | 0.99 | 22.3 ± 2.4 | 22.8 ± 2.4 | 23.4 ± 3.8 | 0.22 |
| Gestational age at enrollment, wk | 16.8 ± 2.1 | 17.0 ± 2.1 | 0.43 | 16.8 ± 2.1 | 16.8 ± 2.1 | 16.8 ± 2.2 | 0.97 | 16.4 ± 2.1 | 16.9 ± 2.3 | 17.2 ± 2.1 | 0.76 |
| Maternal education, completed years | 4.0 ± 3.4 | 4.3 ± 3.6 | 0.40 | 3.9 ± 3.4 | 4.0 ± 3.4 | 4.1 ± 3.6 | 0.65 | 4.4 ± 3.6 | 4.6 ± 3.9 | 4.0 ± 3.4 | 0.84 |
| Proportion of nulliparous women, % | 20.5 | 34.1 | **0.001** | 18.9 | 21.4 | 21.3 | 0.59 | 34.9 | 36.7 | 30.2 | 0.85 |
| Proportion of anemic women (Hb < 100 g/L), % | 20.2 | 25.2 | 0.18 | 20.2 | 19.0 | 21.3 | 0.71 | 27.9 | 27.1 | 20.5 | 0.71 |
| Proportion of women with a positive HIV test, % | 13.8 | 12.5 | 0.88 | 15.5 | 11.2 | 14.7 | 0.16 | 17.1 | 10.0 | 9.7 | 0.64 |
| Proportion of women with a positive malaria test (RDT), % | 23.2 | 23.5 | 0.92 | 21.4 | 24.8 | 23.3 | 0.53 | 34.9 | 18.4 | 18.2 | 0.12 |

^a^ IFA, iron and folic acid; LNS, lipid based nutrient supplement; MMN, multiple micronutrients.

^b^ *P* value obtained from ANOVA (continuous variables) or Fisher’s exact test (proportions)

^c^ Mean ± SD (all such values).
