# Supplementary material for: Association between prenatal provision of lipid‐based nutrient supplements and caesarean delivery: Findings from a randomised controlled trial in Malawi
Source: Matern Child Nutr. 2022 Jul 31;18(4):e13414. doi: 10.1111/mcn.13414 (PMC9480947; doi:10.1111/mcn.13414)
Supplement: Supplementary file 3 — Supporting information. [file MCN-18-e13414-s002.docx]

## Supplemental Table 2. Factors associated with emergency cesarean section.

|  | Bivariate analysis^a^ | |  | Multivariable model I^b^ | |  | Multivariable model II^c^ | |  |
| --- | --- | --- | --- | --- | --- | --- | --- | --- | --- |
| Exposure variables | RR (95% CI) | *P* value |  | RR (95% CI) | *P* value |  | RR (95% CI) | *P* value | |
| Group (LNS to IFA)^d^ | 1.9 (1.1, 3.5) | **0.026** |  | 1.9 (1.0, 3.4) | **0.040** |  | 2.1 (1.0, 4.4) | 0.059 | |
| Sex (male) | 1.0 (0.6, 1.6) | 0.910 |  | 1.0 (0.6, 1.5) | 0.853 |  | 0.9 (0.5, 1.7) | 0.842 | |
| Parity (primiparous) | 2.4 (1.5, 3.8) | **<0.001** |  | 1.8 (1.4, 3.6) | **0.002** |  | 2.3 (1.3, 4.2) | **0.005** | |
| Gestational age at birth (GA), wk | 1.1 (1.0, 1.2) | 0.157 |  | 1.1 (1.0, 1.2) | 0.135 |  | 1.2 (1.0, 1.5) | 0.055 | |
| Child length-for-age Z-score (LAZ) | 0.8 (0.7, 1.0) | 0.075 |  |  |  |  | 0.7 (0.5, 1.1) | 0.159 | |
| Child weight-for-age Z-score (WAZ) | 0.9 (0.7, 1.2) | 0.644 |  |  |  |  |  |  | |
| Child weight-for-length Z-score (WLZ) | 1.2 (1.0, 1.6) | 0.086 |  |  |  |  | 1.1 (0.8, 1.4) | 0.745 | |
| Child head circumference Z-score (HCZ) | 1.2 (0.9, 1.6) | 0.114 |  |  |  |  | 1.5 (1.0, 2.2) | **0.047** | |
| Maternal height, cm | 0.9 (0.9, 1.0) | **<0.001** |  | 0.9 (0.9, 1.0) | **<0.001** |  | 0.9 (0.8, 1.0) | **<0.001** | |
| Maternal BMI, kg/m^2^ | 1.0 (0.9, 1.1) | 0.567 |  |  |  |  |  |  | |
| Maternal age, y | 1.0 (1.0, 1.0) | 0.966 |  |  |  |  |  |  | |
| Maternal weekly gestational weight gain, kg | 0.6 (0.1, 6.1) | 0.688 |  |  |  |  | 0.4 (0.0, 6.4) | 0.551 | |
| HIV+ at enrollment | 0.9 (0.4, 1.8) | 0.683 |  |  |  |  |  |  | |
| Positive malaria test (RDT) at enrollment | 1.2 (0.7, 2.0) | 0.506 |  |  |  |  |  |  | |
| High AGP (>1) at enrollment | 0.8 (0.4, 1.7) | 0.512 |  |  |  |  |  |  | |
| High CRP (>5) at enrollment | 0.7 (0.4, 1.1) | 0.112 |  | 0.7 (0.4, 1.2) | 0.188 |  | 0.6 (0.3, 1.2) | 0.155 | |
| Maternal education, completed years at school | 1.1 (1.0, 1.1) | **0.019** |  | 1.1 (1.0, 1.1) | **0.018** |  | 1.1 (1.0, 1.2) | 0.094 | |
| Household asset, below median | 0.7 (0.4, 1.1) | 0.088 |  |  |  |  |  |  | |

^a^ Log-binomial regression, non-adjusted models. Variables individually compared to emergency caesarean section. N for individual variables ranged from 1015 to 1255.

^b^ Log-binomial regression, adjusted for group, sex, primiparity, GA, maternal height, CRP, and maternal education. N = 1221 for each model.

c Log-binomial regression, adjusted for group, sex, primiparity, GA, maternal height, maternal weekly gestational weight gain, CRP, maternal education, LAZ, WLZ, and HCZ. N = 1030.

d IFA, iron and folic acid; LNS, lipid based nutrient supplement; RR, relative risk, change of one unit for continuous variables and comparison to an opposite value for binary variables.
