# Supplementary material for: Association between prenatal provision of lipid‐based nutrient supplements and caesarean delivery: Findings from a randomised controlled trial in Malawi
Source: Matern Child Nutr. 2022 Jul 31;18(4):e13414. doi: 10.1111/mcn.13414 (PMC9480947; doi:10.1111/mcn.13414)
Supplement: Supplementary file 4 — Supporting information. [file MCN-18-e13414-s003.docx]

## Supplemental Table 3. Stratified analysis of emergency caesarean section between groups^a^

|  |  |  | Emergency caesarean section by study group | | | |  | Comparison between LNS and IFA group^c,d^ | | Comparison between MMN and IFA group^c,d^ | |
| --- | --- | --- | --- | --- | --- | --- | --- | --- | --- | --- | --- |
| Maternal characteristics | Interaction test *P* value^d^ | Split | IFA | MMN | LNS | *P* value^b^ |  | RR  (95 % CI) | *P* value | RR  (95 % CI) | *P* value |
| Parity | **0.071** | Primiparous | 12.7%  (10/79) | 7.9%  (7/89) | 10.1%  (9/89) | 0.553 |  | 0.9  (0.4, 2.0) | 0.717 | 0.6  (0.3, 1.6) | 0.349 |
|  |  | Multiparous | 1.8%  (6/340) | 4.3%  (14/327) | 6.7%  (22/329) | **0.005** |  | 3.6  (1.5, 8.9) | **0.005** | 2.2  (0.8, 5.7) | 0.105 |
| Malaria | 0.131 | Malaria­– | 2.7%  (9/330) | 4.8%  (15/313) | 8.2%  (26/319) | **0.008** |  | 2.8  (1.3, 5.8) | **0.007** | 1.5  (0.7, 3.5) | 0.299 |
|  |  | Malaria+ | 7.8%  (7/90) | 5.8%  (6/103) | 5.2%  (5/97) | 0.789 |  | 0.7  (0.2, 2.3) | 0.563 | 0.8  (0.3, 2.3) | 0.611 |
| Education | **0.092** | Education in years below median | 4.3%  (9/210) | 4.5%  (9/200) | 3.9%  (8/205) | 0.968 |  | 0.9  (0.3, 2.2) | 0.769 | 1.0  (0.4, 2.4) | 0.936 |
|  |  | Education in years above or at median | 2.9%  (6/205) | 5.2%  (11/212) | 10.5%  (22/210) | **0.005** |  | 3.4  (1.4, 8.3) | **0.006** | 1.8  (0.7, 4.6) | 0.258 |
| BMI | 0.149 | BMI below median | 2.3%  (5/216) | 6.0%  (13/217) | 7.9%  (16/203) | **0.028** |  | 3.2  (1.2, 8.6) | **0.022** | 2.4  (0.9, 6.7) | 0.084 |
|  |  | BMI above or at median | 5.4%  (11/204) | 4.0%  (8/200) | 7.0%  (15/215) | 0.419 |  | 1.2  (0.6, 2.7) | 0.579 | 0.7  (0.3, 1.7) | 0. 372 |

^a^ IFA, iron and folic acid; LNS, lipid based nutrient supplement; MMN, multiple micronutrients; RR, relative risk.

^b^ *P* value obtained from Fisher’s exact test.

^c^ Relative risk (RR) and *P* value obtained from log-binomial regression.

^d^ All interaction test and stratified analyses were adjusted for binary variables malaria, parity, education, BMI and four-categorial site (hospitals and health clinics).
